# Inactivated Enterovirus 71 Vaccine Produced by 200-L Scale Serum-Free Microcarrier Bioreactor System Provides Cross-Protective Efficacy in Human SCARB2 Transgenic Mouse

**DOI:** 10.1371/journal.pone.0136420

**Published:** 2015-08-19

**Authors:** Chia-Ying Wu, Yi-Wen Lin, Chia-Ho Kuo, Wan-Hsin Liu, Hsiu-Fen Tai, Chien-Hung Pan, Yung-Tsung Chen, Pei-Wen Hsiao, Chi-Hsien Chan, Ching-Chuan Chang, Chung-Cheng Liu, Yen-Hung Chow, Juine-Ruey Chen

**Affiliations:** 1 Adimmune Corporation, Taichung, Taiwan; 2 Institute of Infectious Disease and Vaccinology, National Health Research Institutes, Zhunan, Miaoli County, Taiwan; 3 Agricultural Biotechnology Research Center, Academia Sinica, Taipei, Taiwan; 4 Enimmune Corporation, Taichung, Taiwan; 5 Graduate Institute of Immunology, China Medical University, Taichung, Taiwan; Nanyang Technological University, SINGAPORE

## Abstract

Epidemics and outbreaks caused by infections of several subgenotypes of EV71 and other serotypes of coxsackie A viruses have raised serious public health concerns in the Asia-Pacific region. These concerns highlight the urgent need to develop a scalable manufacturing platform for producing an effective and sufficient quantity of vaccines against deadly enteroviruses. In this report, we present a platform for the large-scale production of a vaccine based on the inactivated EV71(E59-B4) virus. The viruses were produced in Vero cells in a 200 L bioreactor with serum-free medium, and the viral titer reached 10^7^ TCID_50_/mL 10 days after infection when using an MOI of 10^−4^. The EV71 virus particles were harvested and purified by sucrose density gradient centrifugation. Fractions containing viral particles were pooled based on ELISA and SDS-PAGE. TEM was used to characterize the morphologies of the viral particles. To evaluate the cross-protective efficacy of the EV71 vaccine, the pooled antigens were combined with squalene-based adjuvant (AddaVAX) or aluminum phosphate (AlPO_4_) and tested in human SCARB2 transgenic (Tg) mice. The Tg mice immunized with either the AddaVAX- or AlPO_4_-adjuvanted EV71 vaccine were fully protected from challenges by the subgenotype C2 and C4 viruses, and surviving animals did not show any degree of neurological paralysis symptoms or muscle damage. Vaccine treatments significantly reduced virus antigen presented in the central nervous system of Tg mice and alleviated the virus-associated inflammatory response. These results strongly suggest that this preparation results in an efficacious vaccine and that the microcarrier/bioreactor platform offers a superior alternative to the previously described roller-bottle system.

## Introduction

Enterovirus 71 (EV71) is one of the major pathogens for hand-foot-and-mouth disease (HFMD), which is sometimes associated with severe neurological complications in young children leading to poliomyelitis-like paralysis, meningitis, brain stem encephalitis, and even death [1,2,3,4,5,6,7,8]. EV71-related outbreaks occurred in Malaysia in 1997, Taiwan in 1998, and China in 2008, resulting in high fatality rates and raising serious public health concerns [1,2]. In recent years, most outbreaks in the Western Pacific region are caused by several subgenotypes of EV71 (B3, B4, B5, C1, C2, and C4) virus, which are frequently found to co-circulate with other serotype enteroviruses, such as the coxsackie A virus (CAV), another major causative agent of HFMD [9,10,11,12,13]. This co-circulation increases the potential of genetic recombination among enteroviruses [10,12,14,15]. In fact, the genotypic or serotypic changes in EV71 and CAV have been observed before and may have led to the emergence of novel strains [12,14,15]. Over the last decade, more than 7 million cases of HFMD and 2713 associated deaths have been reported globally [8,16] (http://www.chinacdc.cn/tjsj/fdcrbbg/). No effective drug or vaccine is available for this lethal disease up to this point. Therefore, the development of an effective vaccine to control EV71 epidemics and prevent potential outbreaks is urgently needed.

Recently, clinical trials based on the formalin-inactivated EV71 vaccines have been described by several companies and organizations in Asia. Those vaccines were produced by either roller-bottle or cell-factory technologies with or without serum [13]. Among them, three clinical trials of EV71 vaccines with the subgenotype C4 virus have been independently evaluated in China. In those studies, 30,000 young children and infants were enrolled in each vaccination program, and the results showed that the trial vaccines are safe and efficacious [17,18,19,20]. In Taiwan, an EV71 vaccine derived from the B4 subgenotype has completed human phase I clinical trial by the Vaccine R&D Center of the National Health Research Institutes (NHRI) [21]. Inviragen Pte. Ltd. of Singapore also completed phase I clinical trial with an EV71 vaccine against the B2 subgenotype (http://prsinfo.clinicaltrial.gov/ct2/show/nct01376479?term=inviragen+%28singapore%29+pte+ltd.&rank=12013). The clinical evidence so far supports that inactivated EV71 viral particles may be a potential vaccine candidate for young children and infants.

Recent clinical surveys showed that the vaccine-induced humoral immunity significantly declined after 6 months [22]. Thus, to maintain sufficient neutralizing titers against the EV71 virus, multiple immunizations of the EV71 vaccine may be required for long-term protection [23]. Moreover, several reports indicate that the antibodies elicited by the current EV71 vaccine do not cross-react with CAV16, which is the most common infectious agent that causes HFMD [21,23]. Thus, developing a multivalent vaccine may be necessary to effectively eradicate the epidemics and outbreaks of HFMD [23,24,25,26,27]. All of the above concerns highlight a necessity for the production of several vaccines in sufficient quantity to meet potential demand.

In the previous report, a 40 L scale roller-bottle housed in a 7,500 sq. ft. GMP-certified manufacturing plant produced approximately 50,000 doses (1 μg/per dose) of an EV71 vaccine based on Vero cell culture grown in serum-free media [28,29]. However, the same space can house a 200 L bioreactor, which may result in a five-fold increase in productivity. Furthermore, the bioreactor is a scalable technology platform that may better satisfy the need for producing more EV71 vaccine or multivalent HFMD vaccines. Among all current bioreactor platforms, the microcarrier-based suspension culture combined with single use bioreactor technology can reduce facility complexity and readily expand up to 2,000 L per batch if necessary. Adherent Vero cells cultivated on microcarriers in the bioreactor systems have also been reported to result in 10-fold increased total cell numbers than Vero cells grown in a roller-bottle system [30,31,32,33]. Therefore, this platform has great potential in meeting the surge demand for large quantities of vaccines toward controlling EV71 and HFMD-related diseases.

The complete genome analysis of various EV71 viruses showed the presence of high sequence diversity, which is presumably caused by genetic mutations or recombination among co-circulating viruses [14,34]. Thus, understanding whether the neutralizing antibodies elicited by one subgenotype can be used against viruses of other subgenotypes is important. Recent reports showed that the neutralizing antibody elicited by a vaccine of the C4 strain can cross react with the C2, C4, C5, B4 and B5 subgenotypes [35], whereas a subgenotype B4 vaccine (FI-E59) also elicits neutralizing antibodies against the B1, B4, B5, and C4a but not the C2 subgenotype or CAV [21]. This implies that different subgenotypes of EV71 genotypes may share similar epitopes, but the efficacy of cross-strain neutralization may differ depending on the immune response induced by a selected vaccine strain.

In this study, a 200 L serum-free microcarrier/bioreactor system was established and optimized for the large-scale production of an EV71(E59-B4) vaccine in a CGMP facility. To examine the vaccine efficacy, the immunogenicity and cross-protective potential of bioreactor-produced EV71 vaccines with various formulations were evaluated, and the immune-associated inflammatory responses with vaccination were investigated in human SCARB2 transgenic mice. All results provided compelling evidence that this microcarrier-based suspension bioreactor in a scalable platform offers a good approach to prepare effective and sufficient EV71 vaccine for human use.

## Materials and Methods

### Ethics statement

All animal experiments were conducted in accordance with the guidelines of the Laboratory Animal Center of the National Health Research Institutes (NHRI) in Taiwan. The animal use protocols were reviewed and approved by the NHRI Institutional Animal Care and Use Committee (approved protocol no. NHRI-IACUC-101006-A). The EV71 5746-TW98 (C2) and N3340-TW-02 (C4) strains elicit a lethal neurological virulence of hind limb paralysis (HLP) in human SCARB2 transgenic mice [36]. In the protection of EV71 viral challenge, survival rate was used as one of end points to assess the protective efficacy of EV71 vaccines. Survival rate used as an index of pathogenesis of EV71 infection in experimental animal models has been reported by many studies [36,37,38,39]. All of the tested animals were euthanized by 100% CO_2_ inhalation for 5 minutes followed by cervical dislocation to minimize the animals suffering after the completion of the experimental protocol or when weight loss of 20% occurred (according to the guidelines of approved animal use protocols; protocol no. NHRI-IACUC-101006-A). To perform virus challenge, mice are placed in the anesthetic inhalator chamber containing isoflurane (Initial phase: 5% Maintained phase: 1.5%~2.5%) for 1 minute before subcutaneously injected with EV71 viruses.

### Cell and virus banks

The master Vero cell stocks and CGMP-certificated working virus bank (WVB) of the EV71(E59-B4) strain were obtained via technology transfer from the National Health Research Institutes (NHRI) in Taiwan [40]. The working Vero cell bank (WCB) was delegated and established by BioReliance (UK) following CGMP guidelines for the manufacture of biopharmaceutical products [40].

### Production scale-up of Vero cells and EV71 virus

One vial of WCB was thawed and cultured in a T-flask using VP-SFM (GIBCO) media. The cells were detached by treatment with TrypLE Select (GIBCO) and were transferred to roller bottles (Corning). The 8 roller bottles of harvested cells were seeded into four 3-L spinner flasks containing 3 g/L of microcarrier (Cytodex-1, GE). Vero cells were grown on Cytodex-1 and stirred at 25–40 rpm in 37°C at 5% CO_2_ in an incubator. After the TrypLE Select treatment, the mixture of cells and microcarriers was transferred to a single-use 50 L bioreactor (Sartorius) operated under the conditions of 30 rpm, 37°C, pH 6.8–7.2, and DO 30%. To facilitate high cell density over microcarriers and cell attachment in a serum-free condition, the cell culture was maintained with 25 L of VP-SFM and was then fully filled after cell adherence. When the cell density exceeded 1.0 × 10^6^ cells/mL, the microcarriers were allowed to settle by stopping the rotation, and the culture medium was removed. After the TrypLE Select treatment, the mixture of cells and microcarriers was transferred to a 200 L bioreactor (Sartorius) and cultured at 20 rpm, 37°C, pH 6.8–7.2, and DO 30% until the cell density reached 1 × 10^6^ cells/mL. Subsequently, the microcarriers were allowed to settle and 100 L of conditional medium was removed, and then the bioreactor was operated at 20 rpm, 37°C, pH 6.8–7.2, and DO 30% for virus production. To produce EV71 virus, one vial of the WVB thawed in VP-SFM was inoculated into the cells in the bioreactor at a multiplicity of infection (MOI) of 10^−4^, and the bioreactor was continuously operated at controlled conditions for 1 h to facilitate the adsorption of EV71 to the cells. Then, the cells/virus culture was fully filled with VP-SFM up to 200 L and further incubated for another 9–10 days. The endpoint of EV71 culture was when the virus growth plateaued as observed by the sandwich ELISA method and 95% of cells exhibited the cytopathic effect (CPE). The virus titers were determined by a tissue culture infectious dose (TCID_50_) assay based on Vero cells, according to the Reed–Muench method [41].

### Virus purification and inactivation

The virus culture supernatant was clarified using a 4-μm filter (Sartoclear, 293C8HP13AFFF) and a 0.45/0.22-μm filter (Sartopore2, 5445307H7—00) to remove the cell debris, and the filtrate was then concentrated approximately 50-fold with a 100 kDa Polyethersulfone ultrafiltration cassette (Sartocon PESU Cassette, 3021466807E—SG) to remove the low-molecular weight impurities. The final concentrate was passed through an ion exchange column and was then treated with Benzonase to remove the host cell DNA. The purified virus solution was applied on to a 2–55% sucrose density gradient and centrifuged at 35,000 rpm, 4°C, for 30 min using a Core (H) rotor in the HimacCC40 ultracentrifuge (Hitachi Koki). Fractions were collected and measured for sucrose concentration and specific EV71-unit (U/mL) by sandwich ELISA. The pooled virus was dialyzed against PBS using a 100 kDa PESU cassette and was finally sterilized with a 0.22-μm filter. For virus inactivation, a final formalin concentration of 0.025% was used, and the mixture was incubated at 37°C for 6 days. After dialysis with PBS, the inactivated virus preparation was confirmed for loss of infectivity in human rhabdomyosarcoma (RD) cells as described previously [29].

### Electron microscopy of the EV71 vaccine

A sample of 1–2 μg of inactivated EV71 virus was placed on a 200-mesh copper grid. The specimen on the grid was stained with 1% uranyl acetate. Micrographs were obtained by electron microscopy (FEG-TEM, FEI Tecnai G2 TF20 S-TWIN).

### Vaccine formulation and animal immunization

To prepare the formulated vaccine, each dose of EV71 antigen was mixed with 300 μg/mL of AlPO_4_ (ADJU-PHOS, BRENNTAG) or AddaVAX in sterile phosphate-buffered saline PBS. AddaVAX (Invivogen) is an oil-in-water emulsion with a formulation similar to the MF59 adjuvant (Novartis). The immune schedules designed for the evaluation of EV71 vaccine efficacy in rabbits, hSCARB2 transgenic (Tg) mice and BALB/c mice are shown in S1 Text. The White New Zealand rabbits (3 months old) were intramuscularly immunized twice at days 0 and 21 and bled on day 35 for serum collection. Tg mice were subcutaneously immunized twice with EV71 vaccines, and the antisera were collected at days 12 or 14 after birth. Groups of BALB/c mice were intramuscularly immunized twice at days 0 and 14 and were bled at days 14 and 28 post-immunization for collection of serum samples.

### Microneutralization

The virus-neutralizing titers of mock and post-vaccination mice and rabbit sera were determined in a modified plaque reduction assay [42]. Briefly, each serum was diluted from 1:8 to 1:32,768 and was co-incubated with an equal volume of distinct viruses at a multiplicity of infection (MOI) of 1. The serum/virus mixtures were incubated at 37°C and 5% CO_2_ for 1 h. Vero cell monolayers (95% confluence), prepared in 96-well plates, were infected with 100 μL/well of the mixture, and viral adsorption was allowed for 1.5 h in the incubator. After incubation, the plates were immediately overlaid with 1.25% Avicel RC591 (kindly provided by FMC BioPolymer) prepared in 1× M199 with 2% FBS. The plates were incubated at 37°C in a 5% CO_2_ incubator for 20 h to allow for plaque formation. Viral plaques were stained with Mab979 antibody (Millipore), and the resulting plaques were counted by eye under the microscope. Microneutralization titers were calculated from the average of triplicate sample wells by extrapolating the inverse dilution of serum that produced a 100% reduction of virus by comparing the total number of plaques revealed in PBS serum and vaccination serum samples.

### ELISpot assay

Spleens harvested from mock-treated and vaccinated BALB/c mice 28 days post-immunization and 12 or 14 days after birth from Tg mice were used to isolate splenocytes for ELISpot assays. The detailed procedures are performed as described previously [36].

### Virus challenge

Clinically isolated strains of EV71, Tainan/5746/98 (C2) (GenBank: AF304457.1), and N3340-TW-02 (C4) (GenBank: EU131776.1) were propagated in Vero cells, and the Tg mice were bred and maintained as previously described [36]. To evaluate vaccine efficacy, Tg mice were vaccinated subcutaneously with various EV71 vaccines or PBS at day 1 and day 8 after birth. A total of 3 × 10^6^ pfu of the EV71 5746 (C2) strain or 1 × 10^6^ pfu of the EV71 3340 (C4) strain were individually injected subcutaneously at day 14 or day 12. Changes in body weight and neurological symptom development were observed in the mice for 15 days post-infection. Groups of mice were sacrificed to collect tissues for real-time RT-PCR and immune-histological experiments at day 6 post-infection. All of the tested animals were euthanized by 100% CO_2_ inhalation for 5 minutes followed by cervical dislocation after completion of the experimental protocol or when weight loss of 20% occurred (according to the guidelines of approved animal use protocols; protocol no. NHRI-IACUC-101006-A).

### H&E and immunohistochemical staining

The tissues from sacrificed Tg mice were fixed with 10% buffered formalin (Sigma-Aldrich) and then embedded in paraffin (Sigma-Aldrich), sectioned, and stained with hematoxylin and eosin (H&E). To detect virus particles in tissues, immunohistochemical staining was performed as previously described [36]. Bright field microscopy pictures were taken at 200X magnification.

### Real-time RT-PCR

Tissues from specific organs were homogenized to extract RNA. The cDNA was synthesized using a HiScript I Reverse Transcriptase (Bionovas) and subjected to quantitative PCR analysis (LightCycler*H*480 SYBR Green Real-Time PCR system) with primer pairs specific to target genes as shown in Table 1. CXCL10, TNF-α, and mouse β-actin were detected with the KAPA SYBR FAST qPCR kit following the instrument. To determine the expression of IFN-γ, a TaqMan assay was performed with specific primers and probe No. 69 from the Universal Probe Library (Cat. No. 04688686001, Roche). The relative expression of the target gene normalized to mouse β-actin gene was calculated as previously described [36].

**Table 1:** List of the sequence of primer pairs specific to target genes.

**mouse CXCL10**	
**F**	CTCTCTCCATCACTCCCCTTTAC
**R**	ACTTAGAACTGACGAGCCTGAGC
**mouse TNF-α**	
**F**	TCTCATGCACCACCATCAAGGACT
**R**	TTGCACCTCAGGGAAGAATCTGGA
**mouse IFN-γ**	
**F**	TCAAAAGAGTTCCTTATGTGCCTA
**R**	TACGAGGACGGAGAGCTGTT
**mouse β-actin**	
**F**	ACCAACTGGGACGACATGGAGAAA
**R**	TAGCACAGCCTGGATAGCAACGTA

F:Forward primer; R:Reverse primer

### Statistical analysis

The significant differences between vaccine groups were statistically computed by applying the *t*-test using GraphPad Prism software, Version 6.0.

## Results

### The EV71 virus is cultured in serum-free medium with 200 L single use bioreactor technology

**Fig 1:**
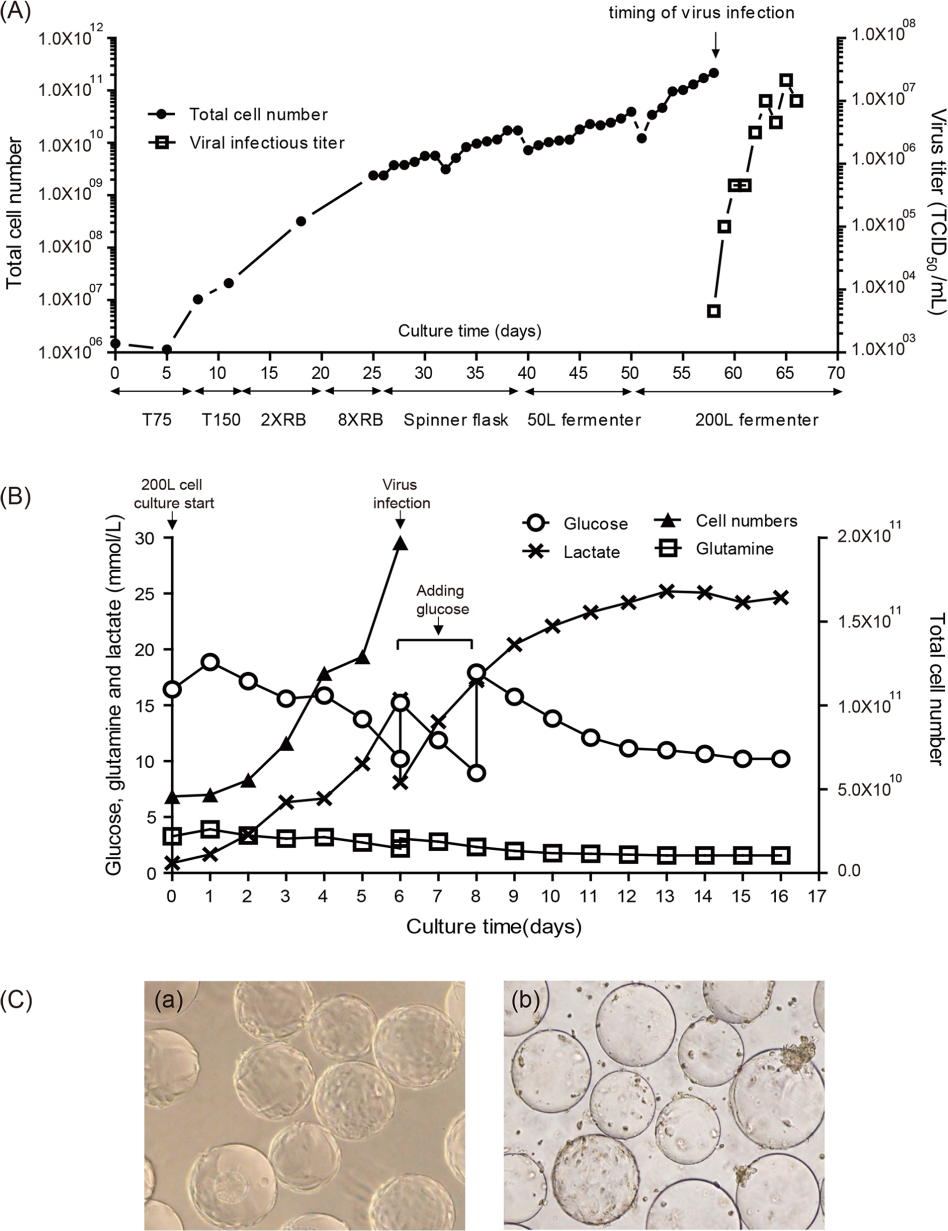
Scale-up of Vero cell culture and EV71(E59-B4) virus production in a 200-L bioreactor. (A) Profile of Vero cell growth and EV71 amplification during a 200-L bioreactor process. (B) Metabolism profile by monitoring the contents of glucose, glutamine and lactate in a 200-L cell/EV71 virus culture. (C) Photomicrographs of Vero cells on microcarriers taken from the 200-L bioreactor immediately before infection with EV71 virus (a) and before harvest (b).

To prepare Vero cells for EV71 virus infection in the bioreactor, the host Vero cells were serially amplified from a single vial to a final 200 L bioreactor using serum-free VP-SFM and TrypLE Select (Invitrogen, animal-component free). A typical profile of cell growth is shown in Fig 1A. The serial cell expansions were performed in T-flasks, roller bottles, spinner flasks, and a 50 L bioreactor. Then, the detached cells/microcarriers mixture from the 50 L bioreactor was transferred into a 200 L bioreactor at an initial cell density of 1 × 10^5^ cells/mL. The cells grew to a cell density of approximately 1 × 10^6^ cells/mL after 6 days (Fig 1A and 1C-a). The virus infection was performed at day 6 with an MOI of 10^−4^. The metabolism profile of cells (glucose, glutamine, and lactate concentration) was monitored daily, and more nutrients were added between days 6 and 8 when maintaining optimal conditions is necessary (Fig 1B). The virus production reached a maximal titer of 10^7^ TCID_50_/mL, and the cells exhibited 95% cytopathic effect at day 10 post-infection (Fig 1A and 1C-b).

### Purification, characterization, and inactivation of the EV71 vaccine

**Fig 2:**
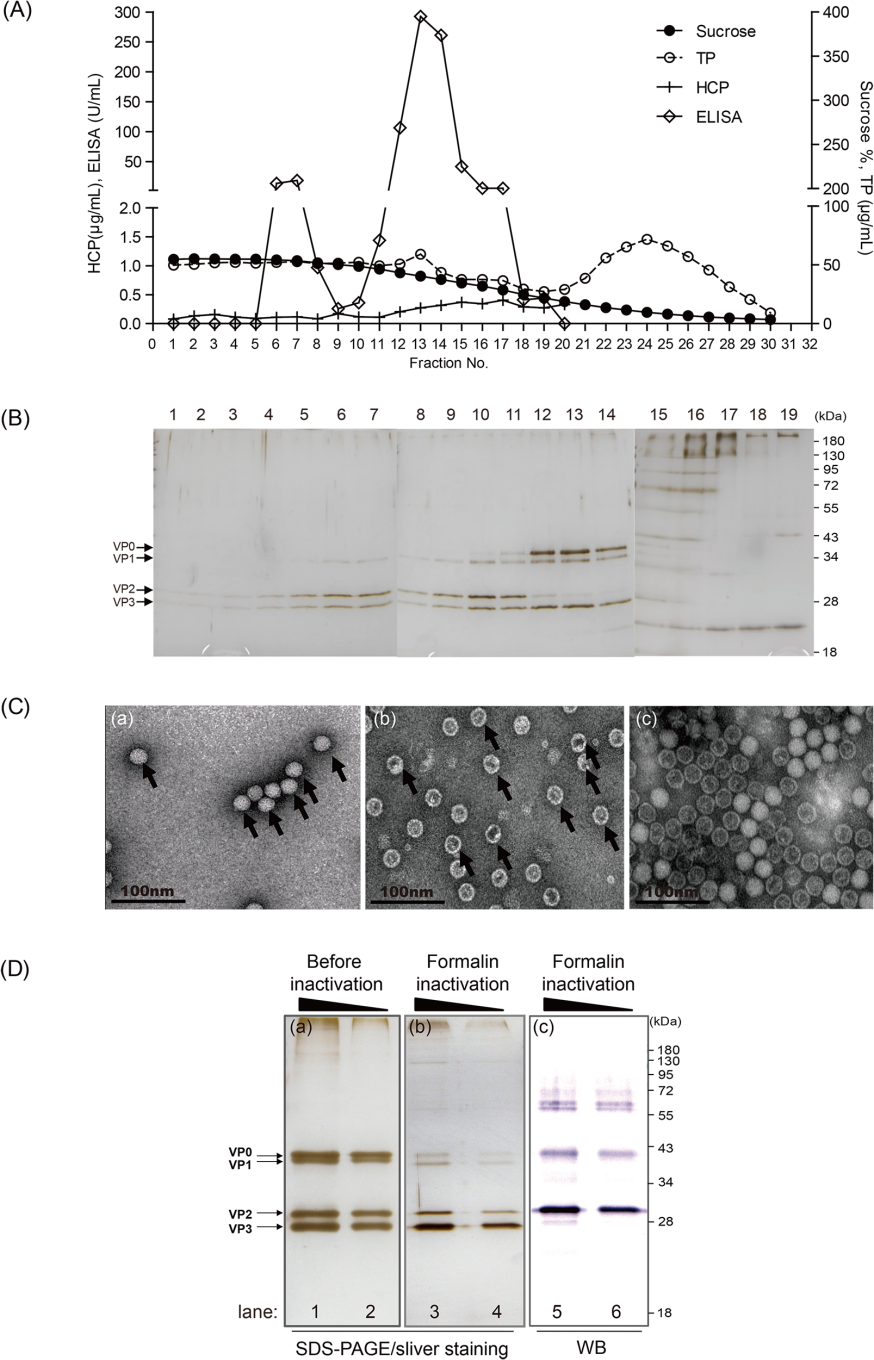
The purification and characterization of the EV71 virus. (A) Purification of EV71 by sucrose density gradient using zonal ultracentrifugation and fractionation analysis by sucrose percentage (Sucrose), contents of total protein (TP) and Vero cell-derived protein (HCP), and EV71-specific units (ELISA). (B) Definition of viral particles distributed in fractions using SDS-PAGE. (C) Electromicrographs of EV71 particles by transmission electron microscopy. (a) Fraction 10 presented a full-particle (F-particle) with infectious capability and a solid particle structure; (b) fraction 13 presented a non-infectious particle (E-particle) with defective particle structure; and (c) pooled viral particles for the vaccine bulk preparation. (D) Quality confirmation of EV71 pooled viral particles before and after formalin inactivation using SDS-PAGE and western blot analysis with a specific monoclonal antibody (Mab979). The molecular weight of VP0, 1, 2, and 3 are as indicated. The black triangle indicates the quantities of loading antigen from high to low.

The harvested viruses were separated from cell debris and microcarriers and were concentrated by a PESU ultrafiltration cassette as described in the Materials and Methods. Subsequently, the concentrated viruses were purified using sucrose density gradient centrifugation. Sucrose density, total protein, ELISA, host cell protein (HCP) and protein profile by SDS-PAGE were performed to analyze the content of every fraction (Fig 2A and 2B). Based on an in-house sandwich ELISA assay, EV71 viral antigens appear to distribute among fractions 5–15 (Fig 2A). SDS-PAGE and silver staining analysis showed several viral antigens (e.g., VP0, VP1, VP2, and VP3) that co-migrated among fractions 5 to 14. Fractions 5–10 and fractions 11–14 from the sedimentation in the sucrose density gradient (Fig 2B) appear to be consistent with the infectious particles (F-particle) and non-infectious particles (E-particle) of EV71 described previously [31]. Negative staining EM was performed to further observe the content of the different fractions. Fractions 5–10 and fractions 11–14 revealed that the physical appearances of viral particles approximately 30–35 nm in diameter were consistent with either full or empty morphologies as observed previously (Fig 2C) [31].

All fractions from 5 to 14 were pooled and treated with formalin (final 0.025%) at 37°C for 6 days. The TCID_50_ of the virus particles fell more than 10^6^-fold below the assay limit while maintaining same antigen concentration by ELISA assay (data not shown). The viral antigens were also found to be extensively cross-linked by the appearance of ultra-high molecular weight proteins on SDS-PAGE after formalin inactivation (Fig 2D, a vs. b). The migration pattern of the major antigens VP0 and VP2 was determined by western blot analysis with a specific antibody (Mab979, Millipore) in inactivated pooled viral particles (Fig 2D-c).

### Immune response to the EV71 vaccine in mice and rabbit

**Fig 3:**
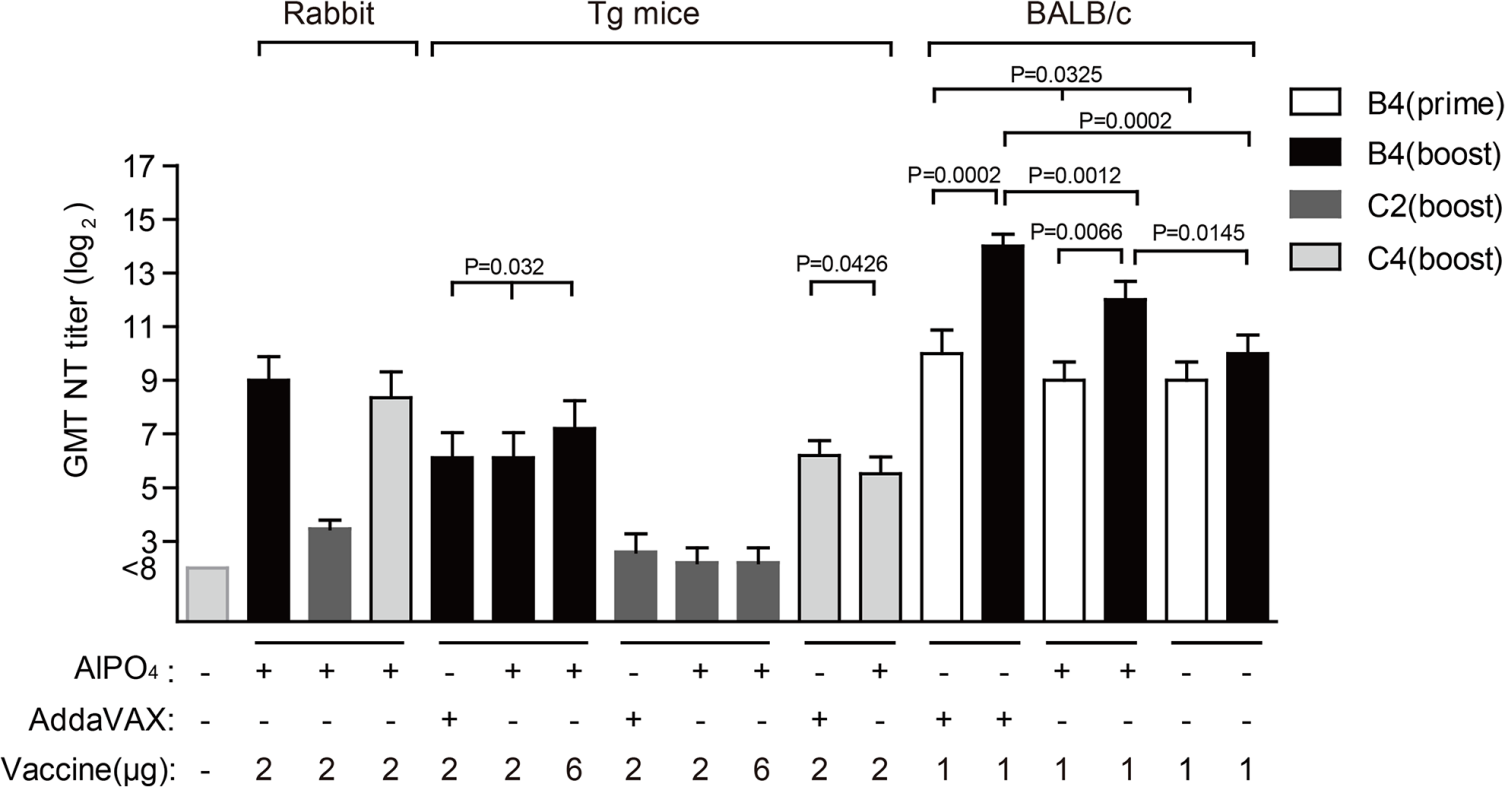
Antibody response to the EV71 vaccine in rabbits, hSCARB2 transgenic (Tg) mice and BALB/c mice. Measurement of vaccine elicited neutralizing antibody titers against EV71 viruses. The used vaccine formulations to immunize animals and the tested subgenotypes of EV71 virus are as labeled. “Prime” or “boost” indicate the detected viral neutralizing titers (NT) in antisera collected from animals that had been immunized once or twice.

**Fig 4:**
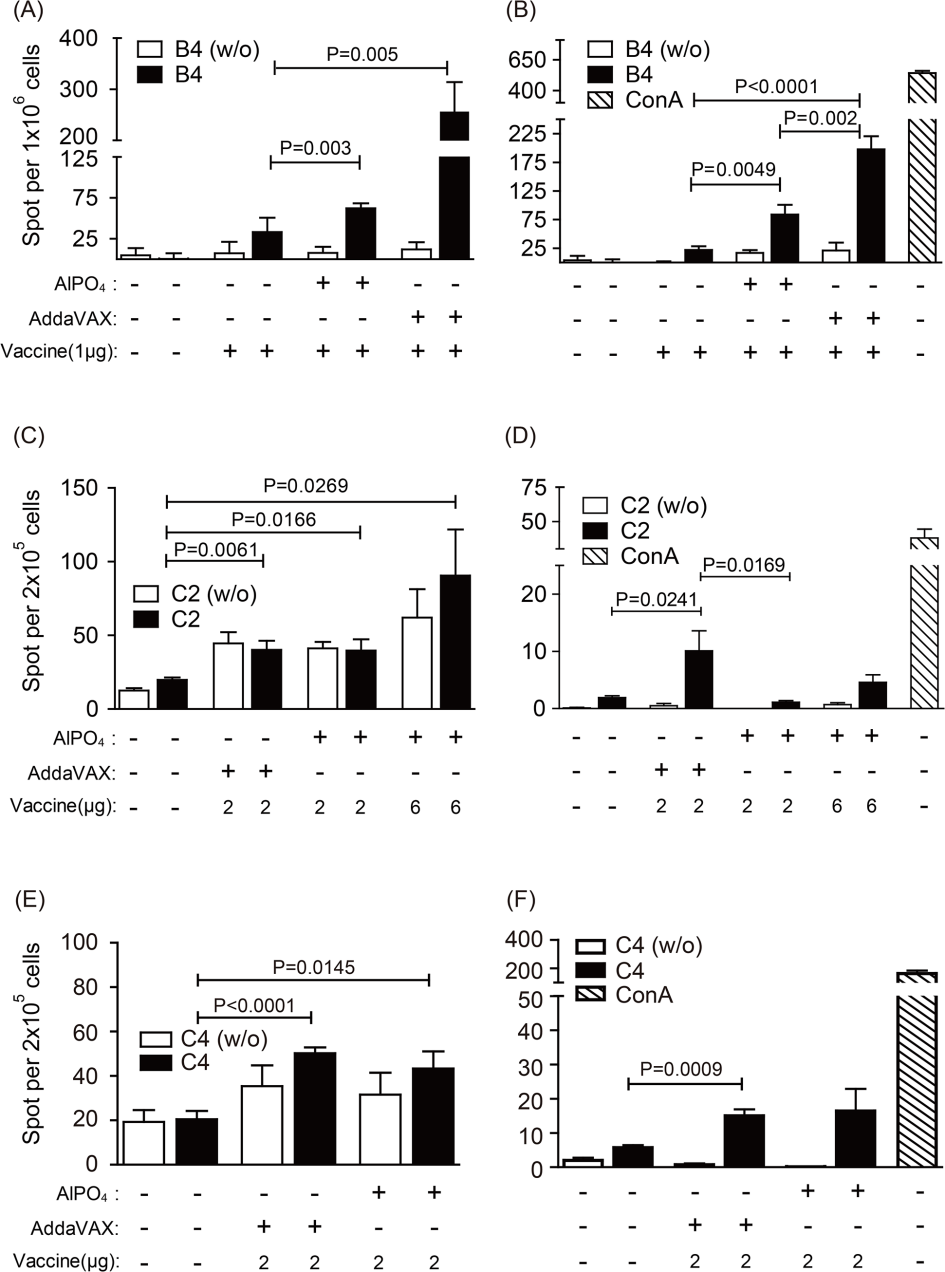
T helper cell responses in vaccinated mice. The levels of IL-4 and IFN-γ spot-forming cells in the spleens of mice were determined by ELISpot assay. Mice groups (n = 5) were immunized with PBS and various dosages of EV71 vaccines including AddaVAX-EV71, AlPO_4_-EV71, or EV71 antigen as indicated. Splenocytes from PBS- or vaccine-treated mice were counted and stimulated *in vitro* with heat-inactivated whole viruses of EV71(E59-B4), 5746 (C2), or 3340 (C4) strains. The cells were stimulated with ConA (1 μg/mL) as a positive control for the IFN-γ ELISpot assay or were mock stimulated as a negative control (w/o) for both ELISpot assays. Bars represent the means ± SEM of spot counts in triplicate wells. The *p* value between vaccine groups relative to the PBS control and two vaccine groups were labeled.

To test the efficacy of the EV71 vaccine produced from the bioreactor system, we investigated the humoral and cell-mediated immune responses elicited by various formulations in BALB/c mice, rabbits, and hSCARB2 transgenic (Tg) mice. Groups of BALB/c mice (6–8 weeks old, n = 5) were immunized twice with various formulations of 1 μg of vaccine antigen at day 0 and 14. The mice antisera were collected for the determination of viral-neutralization titers after 14 and 28 days, and the mice were sacrificed on day 28 post-vaccination to collect splenocytes to examine cell-mediated immune responses (S1 Text). As shown in Fig 3, the neutralizing titer with the sera collected 28 days after immunization was the highest from mice immunized with the AddaVAX-EV71 vaccine (1:16,384) against the EV71(E59-B4) strain compared with sera collected at the same time from mice immunized with the AlPO_4_-formulated vaccine (1:4,096) without adjuvants (1:1,024), and PBS (<1:8). The EV71 antigen without adjuvants exhibited no significant improvement in humoral immune response against the EV71 virus after booster vaccination (Fig 3, prime vs. boost). Both IL-4 and INF-γ were significantly higher in animal groups receiving either AddaVAX- or AlPO_4_-adjuvanted EV71 vaccines than the animal groups receiving antigen alone and PBS (Fig 4A and 4B). All of the above results suggested that the EV71(E59-B4) antigen formulated with adjuvants could achieve both higher humoral and cellular immune responses against homologous virus. To further examine whether the adjuvanted-EV71(E59-B4) vaccines can provide cross-protective immunity against heterotypic strains of EV71, rabbits and Tg mice were vaccinated (S1 Text). Antisera were collected and tested with a cross-strain viral-neutralization assay (Fig 3). The results revealed that the antisera collected from immunized rabbits or Tg mice achieved protective GMT titer ranging from 1:69 to 1:512 against the homotype B4 strain and exhibited significant cross-neutralizing activities against subgenotype 3340 (C4) virus (GMT titer: 1:45 to 1:327). In contrast, 80% of Tg mice vaccinated with the AlPO_4_-EV71 vaccine failed to induce neutralizing antibody to the subgenotype 5746 (C2) strain (GMT titer ≥ 1:8). In the AddaVAX-EV71 group, 60% of Tg mice developed weak cross-neutralizing antibody titers (GMT titer = 1:8) against the C2 strain (Fig 3). Similar results were obtained in rabbits vaccinated with AlPO_4_-formulation with levels of cross-reactive GMT titer (1:8 to 1:16) against the C2 strain (Fig 3).

We next investigated the effect of vaccine formulations on induced cellular immune responses in Tg mice. After vaccination, T-helper cell responses were evaluated by mouse IL-4 (Th2) and IFN-γ (Th1) ELISpot analyses. The results from this type of analysis showed that the level of IL-4 production was higher in animal groups receiving adjuvanted vaccines (only the effect stimulated by heterologous virions was examined); however, the ratio of IL-4 production before and after stimulation by heterologous 5746 (C2) or 3340 (C4) virions exhibit no significant differences between animals receiving either PBS or adjuvanted vaccines (Fig 4C and 4E). Different from situations observed with IL-4, a significantly higher quantity of IFN-γ was observed from the mice vaccinated with the AddaXAV-adjuvanted EV71 vaccine when their splenocytes were stimulated by either heterologous 5746 (C2) or 3340 (C4) viruses (Fig 4D and 4F). These observations suggested that the AlPO_4_-adjuvanted EV71 vaccine induced mostly Th2-mediated humoral immunity, whereas the AddaXAV-adjuvanted EV71 vaccine elicited a balanced Th1/Th2 immunity against EV71 viruses.

### Vaccine treatments protect hSCARB2 transgenic (Tg) mice from heterologous EV71 lethal infection and paralysis

**Fig 5:**
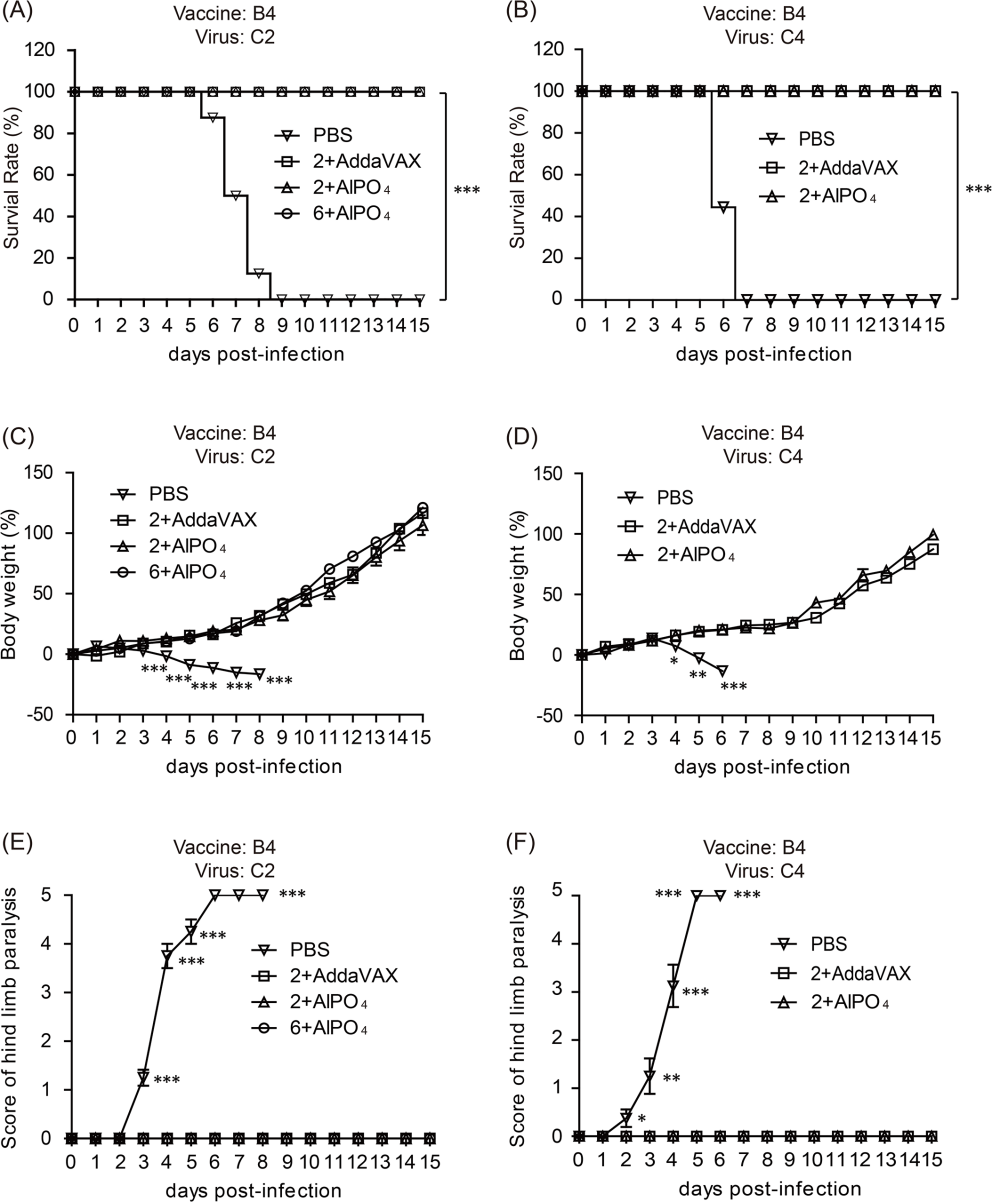
Vaccine treatments protect Tg mice against a lethal-dose challenge of subgenotypes of the EV71 virus. The survival rate of immunized Tg mice challenged with 3×10^6^ pfu of the C2 strain (A) or 1×10^6^ pfu of the C4 strain (B). Mouse body weight change after EV71 C2 (C) or C4 (D) virus challenges. Scores of central nervous system (CNS)-like hind limb paralysis caused by EV71 C2 (E) or C4 (F) viral challenges. The severity of CNS symptoms was scored from 0 to 5 using the following criteria for scoring CNS diseases: 5 = severe front and rear limb paralysis (LP) and no movement, 4 = moderate two rear LP and hesitant movement, 3 = one rear LP with bending legs, 2 = mild rear limb bended, 1 = slightly rear limb bended, and 0 = normal movement. LP is defined as the rigidness of mouse legs that are resist movement. The PBS and vaccination groups are indicated as symbols. *: *p* < 0.05, **：*p* < 0.01, and ***: *p* < 0.001.

**Fig 6:**
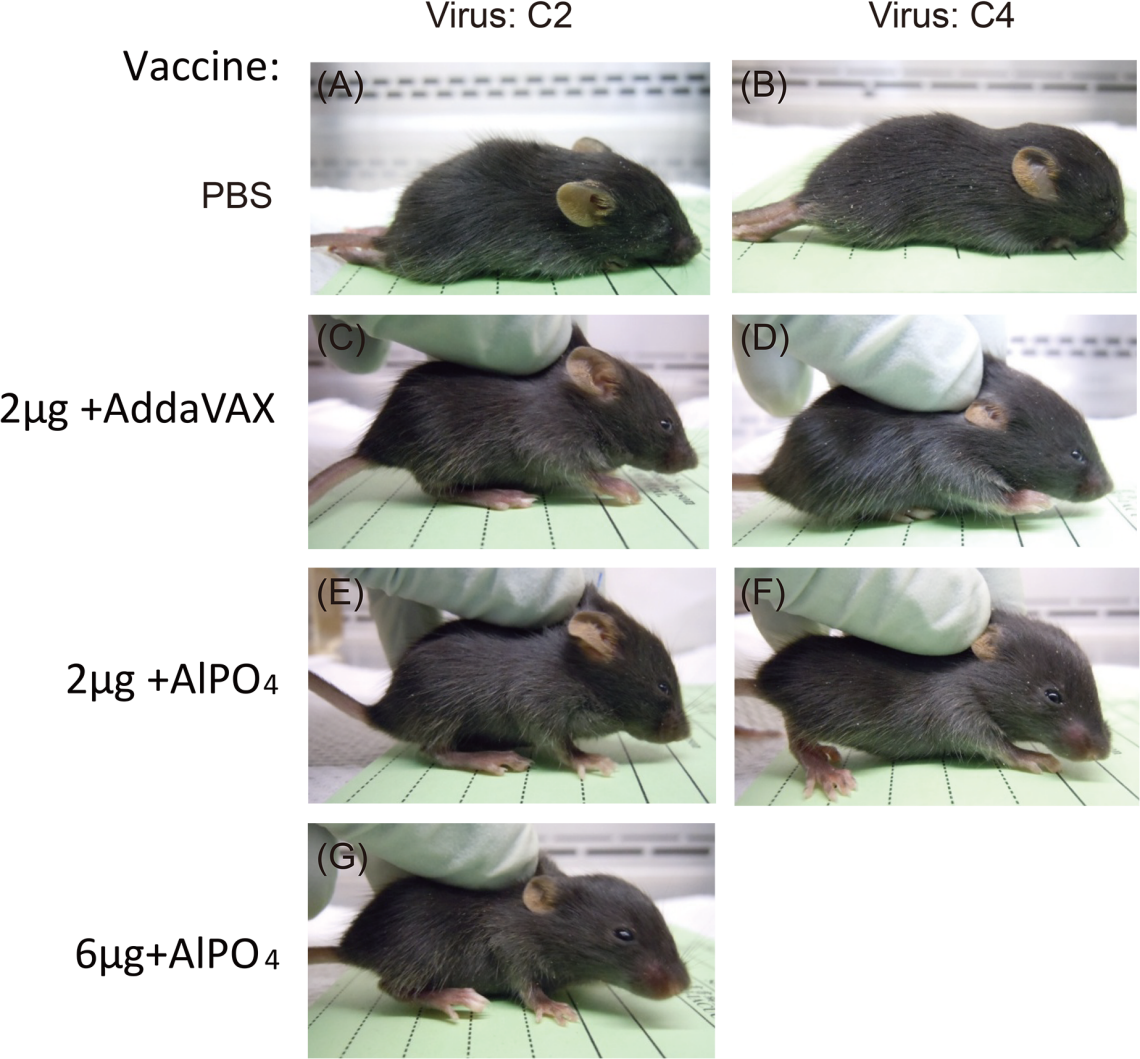
Neurological symptoms in Tg mice after lethal challenge with EV71 C2 or C4 strains. Tg mice receiving PBS (A-B), 2 μg of the EV71 vaccine with AddaVAX (C-D), or 2 μg (E-F) or 6 µg (G) of the EV71 vaccine with AlPO_4_ were challenged with EV71 C2 or C4 viruses. The representative photographs showed the immunized Tg mice at 6 days post-challenge.

**Fig 7:**
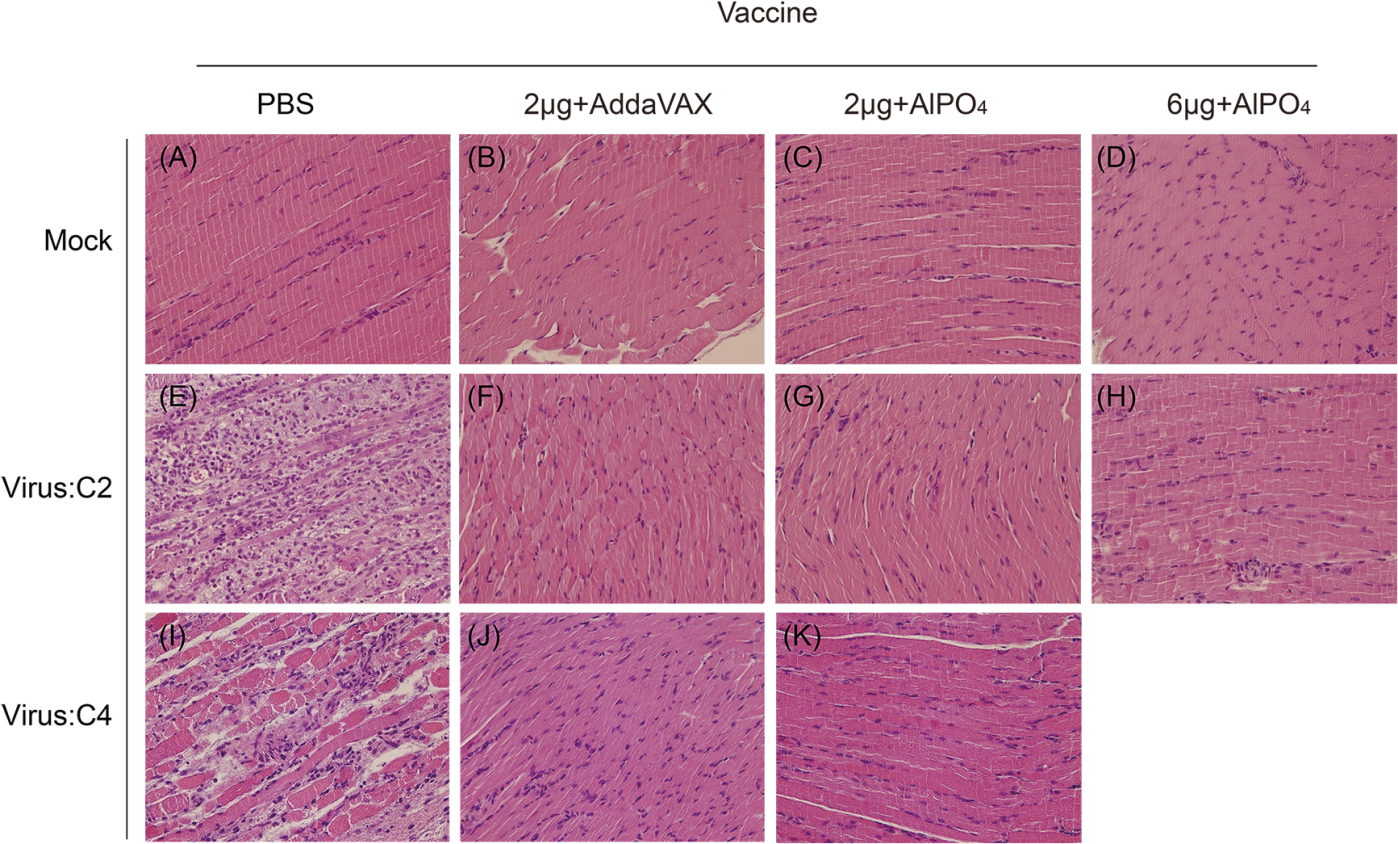
The muscle pathological changes in Tg mice challenged with EV71 viruses. The hematoxylin and eosin (H&E) staining of muscle tissues collected from PBS- or vaccine-treated mice without EV71 challenge (A-D) as control group. H&E staining of muscle tissues collected from PBS- or vaccine-treated mice at 6 days post-infection with the EV71 C2 (E-H) or C4 (I-K) strains. The vaccine formulations and viruses are labeled. All pictures were taken at 200X magnification.

**Fig 8:**
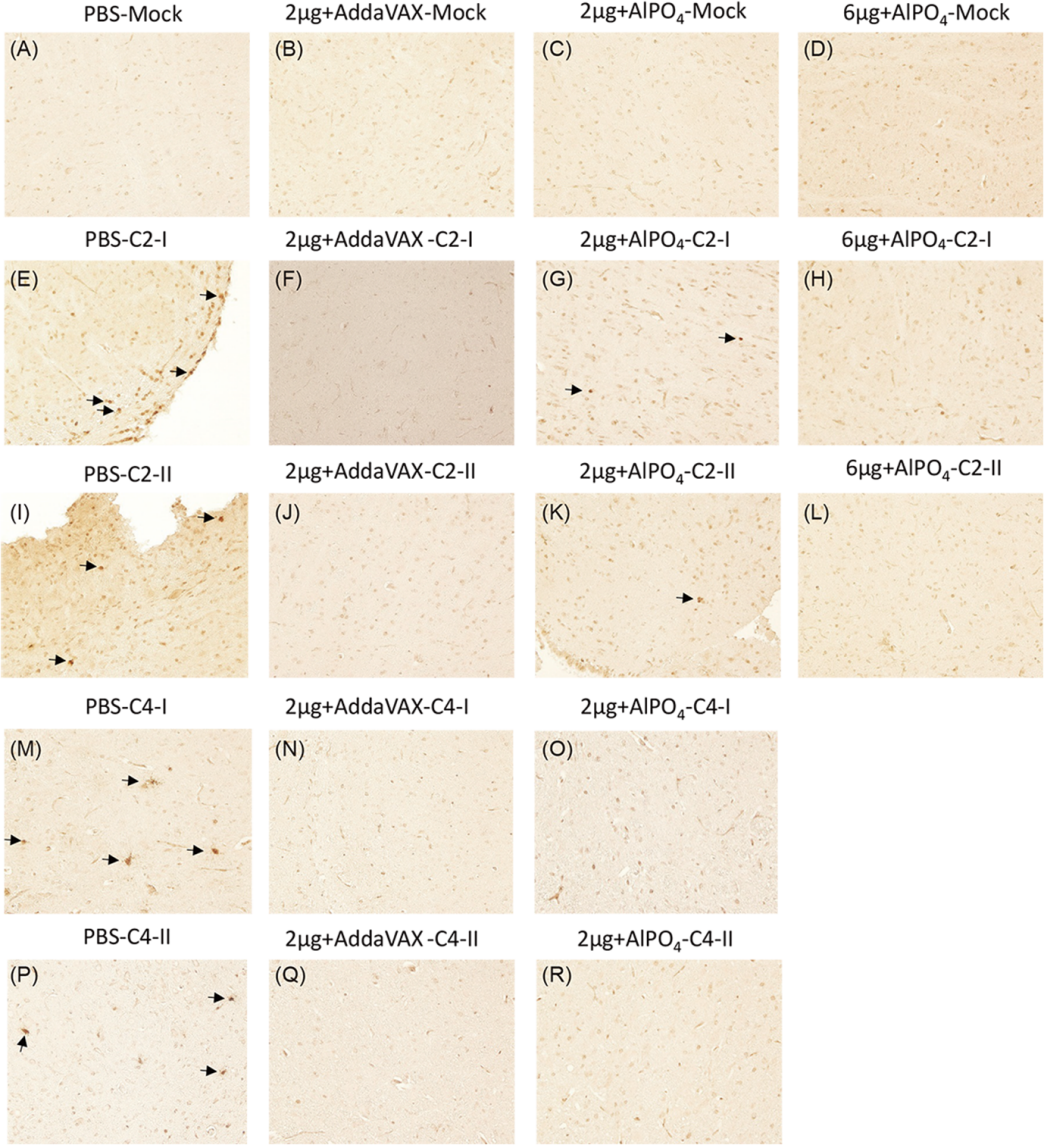
*In situ* detection of EV71 distribution in the brain stems of Tg mice after challenge. The uninfected Tg mice either pre-treated with PBS or vaccines were used as the negative control, and the images of immunohistochemistry (IHC) staining with the Mab979 antibody are shown in A-D. The PBS- and vaccine-treated Tg mice infected with the EV71 C2 or C4 strains were sacrificed on day 6 post-infection, and the waxed sections of brain stem stained by IHC are representatively shown twice in E-R as labeled. All pictures were taken at 200X magnification. Viral particles in the sections are indicated with arrows.

To evaluate the cross-protective efficacy of EV71(E59-B4) antigen combined with adjuvants, the vaccinated Tg mice were challenged with the EV71 5746 (C2) or 3340 (C4) strains as previously described [36]. In contrast to the placebo group (PBS), the different groups of mice vaccinated with the EV71 vaccines were 100% protected against EV71 5746 (C2) and 3340 (C4) virus challenges (Fig 5A and 5B). All mice continually gained weight after different EV71 infections through the 15 days except those that received PBS (Fig 5C and 5D). In addition, the PBS-treated mice exhibited clear central nervous system (CNS) symptoms as early as 2–3 days post-infection (dpi) and peaked on 5–6 dpi depending on the virus strains of infection (Fig 5E and 5F). Representative photographs showed that the PBS-treated mice exhibited serious hind limb paralysis symptoms at 6 dpi, but no apparent symptoms appeared in vaccinated mice (Fig 6). To verify whether the vaccines can effectively prevent EV71-induced neurologic pathogenesis in Tg mice, the muscle and brain stem sections were examined by hematoxylin and eosin and immunohistochemical staining. The results showed clear and well-organized muscle fibers in uninfected mice (Fig 7A–7D, Mock) and vaccinated mice with infections at 6 dpi (Fig 7F–7H and 7J–7K). The inflammatory cells infiltrated into the muscle tissues and the destructed structures were only observed in the PBS group of EV71-infected mice (Fig 7E and 7I). The complete clearance of viral antigen in the brain stem was observed in mice vaccinated with 6 μg of the AlPO_4_-EV71 vaccine or 2 μg of the AddaVAX-EV71 vaccine following EV71 infections (Fig 8F–8Q and 8H–8L). However, numerous loci appeared in the brain stem of PBS-treated mice (Fig 8E–8P), and fewer loci were observed in the brain stem from the mice vaccinated with 2 μg of the AlPO_4_-EV71 vaccine after EV71 5746 (C2) infection (Fig 8G–8K).

### Vaccine treatments significantly suppressed the expression of pro-inflammatory cytokines and chemokines in the tissues of EV71-infected Tg mice

**Fig 9:**
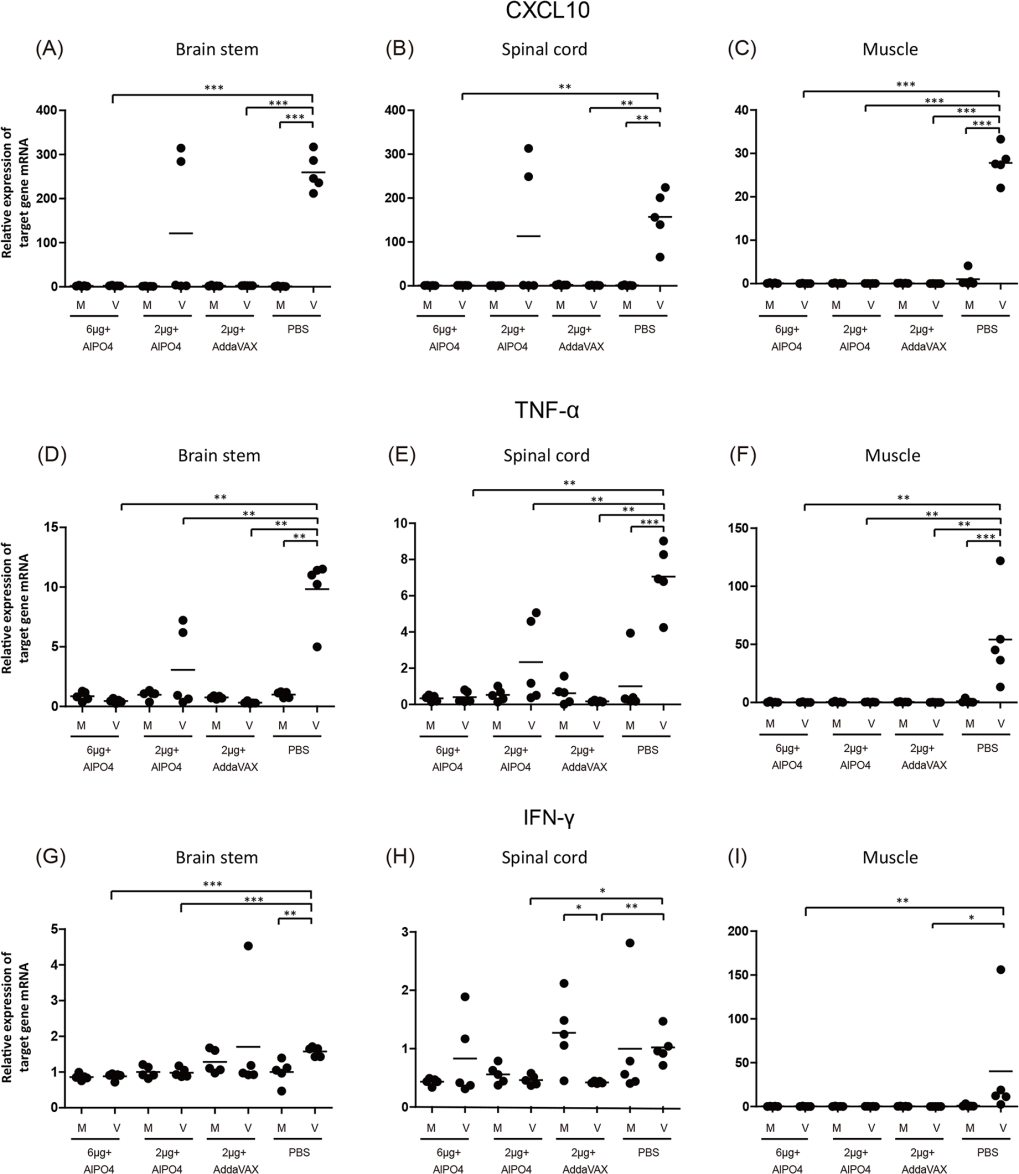
Expression of pro-inflammatory cytokines/chemokines in the CNS compartments and muscle tissue of EV71 (C2)-infected mice. The vaccinated Tg mice were subcutaneously infected with 3×10^6^ pfu of the EV71 5746 (C2) strain, and RNAs were extracted from the brain stem, spinal cord, and muscle at 6 days post-infection. Quantitative RT-PCR analysis was conducted to quantify the gene expression level of CXCL10, TNF-α, and IFN-γ. Unvaccinated and uninfected Tg mice were used as the negative control. The number of PCR cycles required for fluorescent detection of target genes was calculated and presented as the relative expression after normalization with the internal control of β-actin expression from the same tissue. Mock (M): uninfected mice group; Virus (V): EV71 5746 (C2) infected mice group. A schematic representation of the target gene expression and the statistical average from 5 mice per group is shown. The used vaccine formulations are as labeled. Significant difference between each group was shown as *: *p* ﹤ 0.05, **：*p* ﹤ 0.01, and ***: *p* ﹤ 0.001.

**Fig 10:**
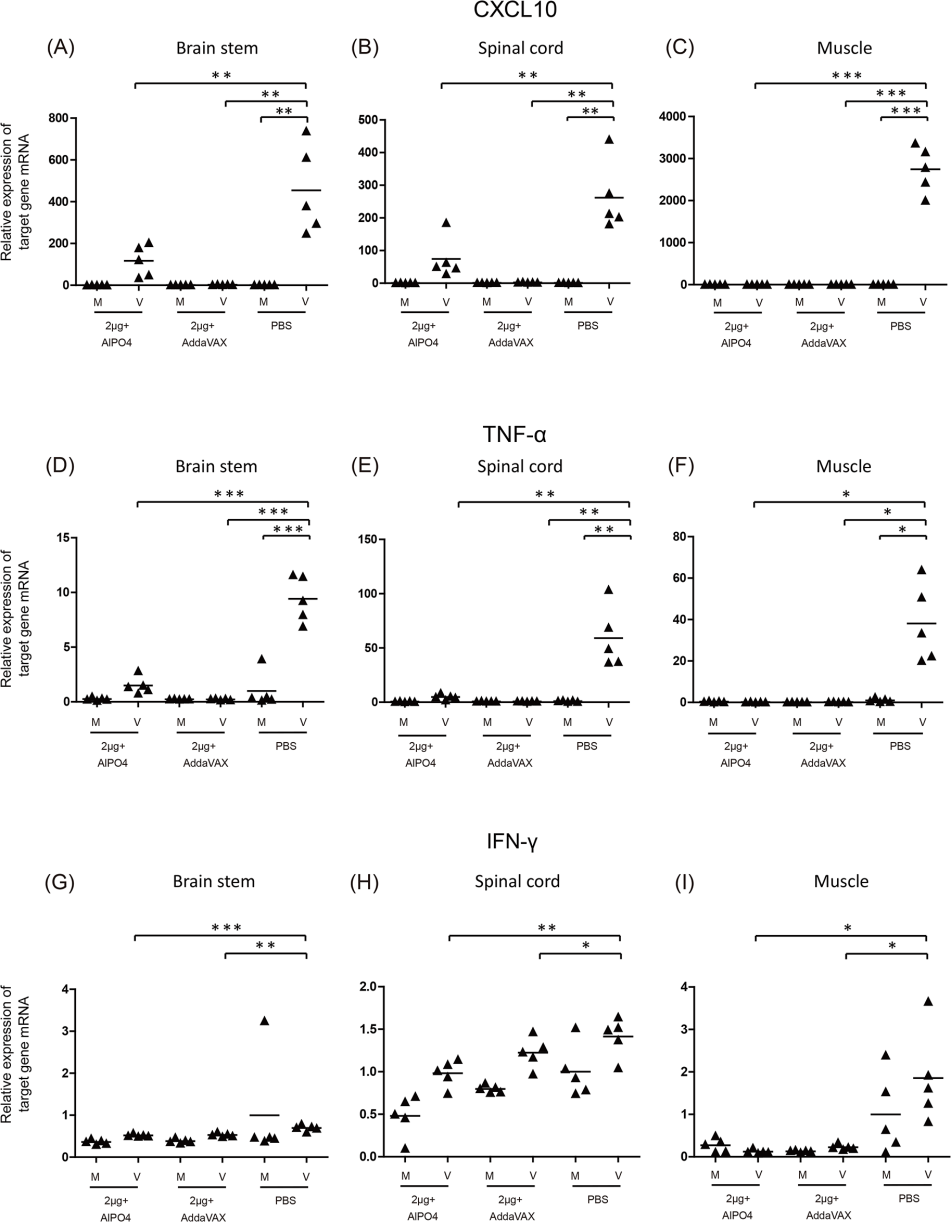
Expression of pro-inflammatory cytokines/chemokines in the CNS compartments and muscle tissue of EV71 (C4)-infected mice. The vaccinated Tg mice were subcutaneously infected with 1×10^6^ pfu of the EV71 3340 (C4) strain, and the RNAs extracted from the brain stem, spinal cord, and muscle at 6 days post-infection were subjected to quantitative RT-PCR analysis. Mock (M): uninfected mice group; Virus (V): EV71 3340 (C4) infected mice group. A schematic representation of the target gene expression and the statistical average from 5 mice per group is shown. The used vaccine formulations are as labeled. Significant difference between each group was shown as *: *p* ﹤ 0.05, **: *p* ﹤ 0.01, and ***: *p* ﹤ 0.001.

Enterovirus infection can induce an extensive inflammatory response in the peripheral and central nervous system, which is responsible for the pathogenesis of EV71-associated disease [43,44,45]. A previous study demonstrated that the hypersecretion of CXCL10, CCL3, TNF-α, and IL-6 correlated to the severity of neurologic pathogenesis induced by EV71 challenge [36]. To confirm whether the vaccine prepared in our platform is able to prevent inflammation-related pathogenesis, the expression of pro-inflammatory indicators were measured after the virus infection in vaccinated Tg mice. As shown in Figs 9 and 10, the pro-inflammatory gene expression of CXCL10, TNF-α, and IFN-γ in the CNS and muscle tissues were up-regulated in the PBS group upon infection with EV71 5746 (C2) or 3340 (C4). In contrast, the expression level of CXCL10, TNF-α, and IFN-γ were significantly lower in the mice vaccinated with either AlPO4- or AddaVAX-EV71 vaccines upon infection with EV71 5746 (C2) or 3340 (C4). In these experiments, 2 μg of the AddaVAX-EV71 vaccine showed equivalent effectiveness as 6 μg of the AlPO_4_-EV71 vaccine, and it was also more potent than 2 μg of the AlPO_4_-EV71 vaccine in inhibiting the inflammatory responses caused by virus infection (Figs 9 and 10). These results suggest that the balance of the Th1/Th2 immune response induced by the AddaVAX-EV71 vaccine provides a role in alleviating the degree of EV71-induced neurologic pathogenesis (Figs 4, 8, 9 and 10).

## Discussion

In this report, we demonstrated a scalable manufacturing system by employing microcarrier-based suspension bioreactor technology for the production of inactivated EV71(E59-B4) vaccine at a 200 L scale with serum-free media (Fig 1). The virus production reaches a titer of 10^7^ TCID_50_/mL, consistent with those exhibited in a roller-bottle system [29]. After purification with sucrose gradient ultracentrifugation (Fig 2), the average total protein yield of purified EV71 vaccine with a B4 subgenotype in the 200 L pilot production scale is approximately 160 mg per batch. Based on our unpublished results, the VP2 epitope content determined by quantitative ELISA in each μg of our purified EV71 antigens is estimated to be approximately 3 to 4 times higher than that described previously [29]. This increase may be due to increased purity resulting from different downstream processes. This production system provides many benefits in the manufacturing of the EV71 vaccine and overcomes the limitations associated with the roller-bottle system. The roller-bottle system is a relatively labor-intensive process, whereas a bioreactor provides a larger vessel per batch for manufacturing and can be readily scaled to a larger volume under standardized conditions with better lot-to-lot consistency. Based on previous publications, a single batch of subgenotype B4 virus manufactured in a 40 L roller bottle system followed with a chromatographic purification process resulted in approximately 50 mg of EV71 vaccine in total protein. Following cGMP regulations, scaling the current 40 L protocol to 200 L would require 3.5 months of overlap manufacturing. In our case, a 200-L scale production system can provide an equivalence of 2 to 3 times more EV71 vaccine within 70 days based on our downstream purification process. Therefore, we believe that the microcarrier/bioreactor platform is a more efficient process when large amounts of enterovirus vaccine are urgently needed.

To examine the biophysical/biochemical and immunological properties of EV71 vaccine prepared with our platform, the vaccine samples were analyzed by SDS-PAGE, TEM, viral-neutralization, and ELISpot assays (Figs 2, 3 and 4). The virus particles thus obtained from the 200 L bioreactor appear to be high in purity and have the structural morphologies consistent with previously published data [31]. The specifications of inactivated EV71 vaccine produced in bulk were also verified to meet the criteria for a human clinical study: the content of Vero cell-derived host cell protein (HCP) was 99.0±13.5 ng/mL and host DNA was at 10±2.5 pg/dose (WHO and US FDA guidelines for the human vaccine: HCP ≤ 4 μg/mL; host DNA ≤ 100 pg/dose).

The immunological results showed that EV71(E59-B4) antigen combined with adjuvants greatly enhance both humoral and cell-mediated immune responses against the EV71 B4 virus in BALB/c mice, suggesting an adjuvant-required role in eliciting a stronger immune response (Figs 3, 4A and 4B). In addition, the antisera from vaccinated rabbits and hSCARB2 transgenic (Tg) mice all exhibited stronger neutralizing antibody titers against homotypic B4 and heterotypic 3340 (C4) viruses but not a 5746 (C2) virus (Fig 3). This result is consistent with previous observations in monkey [46] and a phase I clinical trial [21] with an EV71 vaccine (FI-E59) produced by roller-bottle technology.

The cross-protective potential of bioreactor-produced EV71 antigens combined with AddaVAX and AlPO_4_ adjuvants were also evaluated in a well-established animal model of a Tg mouse [36]. We observed that the Tg mice immunized with both vaccines were fully protected from challenges by C2 and C4 viruses. Pathological observation also showed that vaccinated mice were free of neurological paralysis symptoms associated with viral infection and continued to gain body weight over the experimental period. As a control, the PBS-treated animal group exhibited serious paralysis in the hind limbs and all died before 9 days post-challenge (Figs 5 and 6). Moreover, in the vaccine treated groups, the muscle tissue damage, appearances of viral antigen in the central nervous system (CNS) compartments, and highly regulated pro-inflammatory indicator expression caused by EV71 infection were absent in the animal groups treated by 2 μg of the AddaVAX-EV71 vaccine and 6 μg of the AlPO_4_-EV71 vaccine (Figs 7, 8, 9 and 10). The IL4 and IFN-γ results revealed by the ELISpot assays suggested that the EV71 vaccine with the AddaVAX formulation induced Th1 and Th2 balance immunity which may account for the vaccine’s cross-protective capability at lower doses with significant suppression of EV71-induced neurological symptoms in Tg mice (Figs 4–10). Our results suggest that the squalene-based emulsion is a potential alternative adjuvant comparable to conventional alum adjuvant because it confers a moderate dose-sparing effect and an adequate immune response against distinct EV7 viruses, and similar observations may be validated by human trials.

In conclusion, the results described in this study provide evidence that the inactivated EV71(E59-B4) vaccine could be readily produced from an optimized serum-free microcarrier-based suspension bioreactor platform on a 200 L scale, and we suggest that scale could be increased to 1000 L. The results that the EV71 vaccine also cross-protects against the lethal infection of other EV71 subgenotype strains such as C2 and C4 viruses suggest that this production technology may allow a platform to produce large quantities of protective vaccine if a need arises. Further trials in humans are obviously warranted to fully elucidate the vaccine efficacy and optimal formulation for controlling widespread EV71 and HFMD-related outbreaks.

## Supporting Information

S1 Text The experimental schedules designed for evaluating EV71 vaccine efficacy in animals. (DOC)
