# Supplementary material for: Inactivated Enterovirus 71 Vaccine Produced by 200-L Scale Serum-Free Microcarrier Bioreactor System Provides Cross-Protective Efficacy in Human SCARB2 Transgenic Mouse
Source: PLoS One. 2015 Aug 19;10(8):e0136420. doi: 10.1371/journal.pone.0136420 (PMC4543551; doi:10.1371/journal.pone.0136420)

**Supporting information**

**Figure S1.** The experimental schedules designed for evaluating EV71 vaccine efficacy in rabbits (A), hSCARB2-transgenic (Tg) mice (B), and BALB/c mice (C), respectively. The animals receiving PBS or vaccines were bled according to the time schedule of experimental protocol and the collected serum samples were subjected to microneutralization assay to determine NT titers. The immunized mice were sacrificed to collect the splenocytes for ELISpot assay as labeled. To evaluate the efficacy in protecting of animals against lethal EV71 infection, groups of immunized Tg mice were challenged with the EV71 5746 (C2) or 3340 (C4) viruses following daily monitoring of survival and developed pathological symptom through the 15 days. The tissues of immunized Tg mice were collected at day 6 post-infection for histological and gene expression analyses.


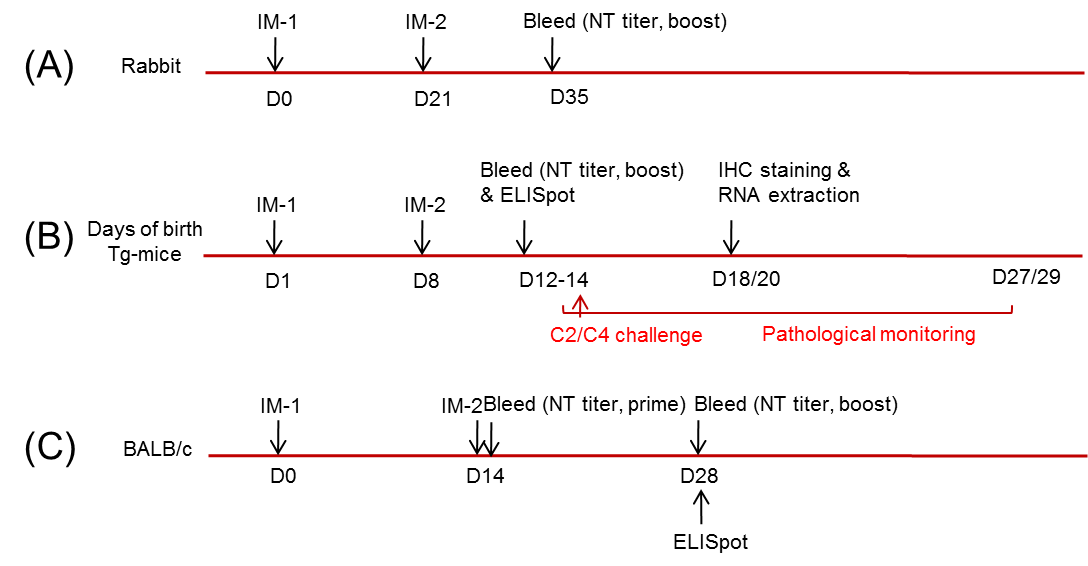

Supplement: S1 Text — (DOC) [file pone.0136420.s001.doc]
